# A Novel Nano-Spherical Tip for Improving Precision in Elastic Modulus Measurements of Polymer Materials via Atomic Force Microscopy

**DOI:** 10.3390/mi15091175

**Published:** 2024-09-22

**Authors:** Tianyu Fu, Paul C. Uzoma, Xiaolei Ding, Pengyuan Wu, Oleksiy Penkov, Huan Hu

**Affiliations:** 1 ZJU-UIUC Institute, International Campus, Zhejiang University, Haining 314400, China; tianyu.21@intl.zju.edu.cn (T.F.); upaul@intl.zju.edu.cn (P.C.U.); xiaolei.22@intl.zju.edu.cn (X.D.); pengyuan.22@intl.zju.edu.cn (P.W.); oleksiypenkov@intl.zju.edu.cn (O.P.); 2 State Key Laboratory of Fluid Power & Mechatronic Systems, Zhejiang University, Hangzhou 310027, China

**Keywords:** atomic force microscopy (AFM), elastic modulus, spherical tip, measurement precision, polymers

## Abstract

Micro-nano-scale mechanical properties are vital for engineering and biological materials. The elastic modulus is generally measured by processing the force–indentation curves obtained by atomic force microscopy (AFM). However, the measurement precision is largely affected by tip shape, tip wear, sample morphology, and the contact model. In such research, it has been found that the radius of the sharp tip increases due to wear during contact scanning, affecting elastic modulus calculations. For flat-ended tips, it is difficult to identify the contact condition, leading to inaccurate results. Our research team has invented a nano-spherical tip, obtained by implanting focused helium ions into a silicon microcantilever, causing it to expand into a silicon nanosphere. This nano-spherical tip has the advantages of sub-micro size and a smooth spherical surface. Comparative tests of the elastic modulus measurement were conducted on polytetrafluoroethylene (PTFE) and polypropylene (PP) using these three tips. Overall, the experimental results show that our nano-spherical tip with a consistent tip radius, symmetrical geometric shape, and resistance to wear and contamination can improve precision in elastic modulus measurements of polymer materials.

## 1. Introduction

AFM has become widely utilized to access the mechanical properties of various material types [1,2,3]. One of the fundamental mechanical properties is elastic modulus, crucial for applications involving engineering materials and biomaterials [4,5,6,7]. AFM enables nanometer-scale measurement of elastic modulus, facilitating a deeper understanding of material properties’ distributions on a nanometer scale. The standard approach to using AFM for elastic modulus is to obtain a force–distance curve by indentation, pressing the AFM tip onto the material and then retracting. Then, with a known tip geometry, the elastic modulus of the sample can be derived from a mechanical model [8,9,10].

Measuring the precise microscopic or submicroscopic elastic modulus has important applications in biological and polymer fields. For example, in Ouyang’s study [11], which used AFM to measure the elastic modulus of subchondral bone slices, the performance of the interface tissue was determined by the mechanical properties in a specific region. Furthermore, AFM has also been also applied to measure the mechanical properties of polymers adjacent to a substrate with nanometer resolution [12]. The application of AFM enables researchers to consistently observe the gradient of mechanical properties away from a substrate, across diverse materials systems. This method paves the way for a profound understanding of the mechanical responses of polymers.

Before the advent of AFM, nano-indentation was extensively employed for material properties testing. It directly measures dynamic contact stiffness during loading and is less affected by thermal drift, ensuring accurate observation of small-volume deformations [13]. The challenge with nanoindentation is that measuring mechanical properties at the submicron and nano levels is tough. As the demand for micro-nano-scale measurements has escalated, AFM has emerged as the premier tool for elastic modulus measurement. Compared with nano-indentation, AFM offers superior control over indentation load, depth, and effective contact area, facilitating precise investigations into the local mechanical behavior of small-scale structures, and it can generate high-resolution maps of elastic modulus [14,15].

The precision of AFM elastic measurements is significantly related to the choice of contact model [10,16,17] and the tip geometry [18,19,20,21,22,23,24,25,26]. The tetrahedral tips commonly used in measurements are prone to wear [18] and contamination [24] during indentation, complicating the estimation of contact area and introducing errors in the fitting model. Additionally, the asymmetry of tetrahedral tips during indentation can lead to anomalous deformation and uneven force distribution [23]. Flat-ended tips also face challenges in examining the contact condition and misalignment between the tip and the sample [25]. Research [19,26] has pointed out that spherical tips can measure mechanical properties more accurately and precisely.

**Figure 1:**
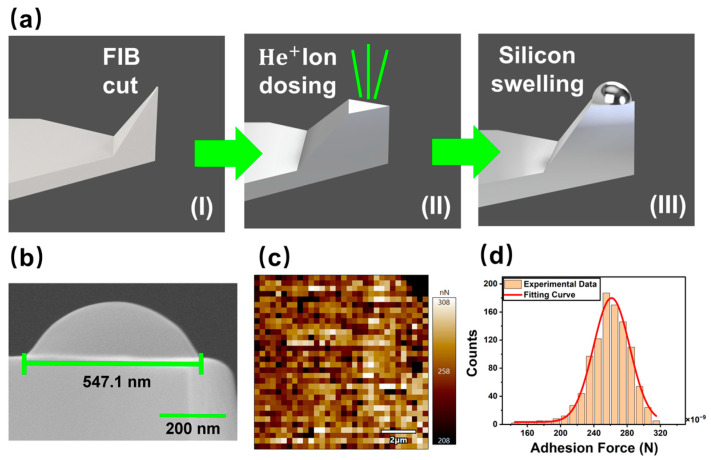
(**a**) Schematic of the manufacturing process of the nano-spherical tip: (**I**) use FIB to cut a sharp tip; (**II**) dose helium ions into the flat-ended tip; (**III**) swell up a nanosphere; (**b**) SEM image of a nano-spherical tip; (**c**) adhesion force map of the surface obtained using the nano-spherical AFM tip; (**d**) Statistical analysis histogram of adhesion forces on a glass substrate.

In this study, a novel nano-spherical tip was applied, to investigate whether it was able to reduce the deviation of elastic modulus measurements. As shown in Figure 1a, this tip was fabricated by implanting focused helium ions into a single-crystal silicon microcantilever to swell up a silicon nanosphere [27]. This is a frequent and undesired phenomenon in HIM (helium ion microscope) imaging, metrology, and nanomachining. The mechanism responsible for this phenomenon is caused by direct momentum transfer between ions and atoms of the irradiated substrate. Under a higher dose (above 1600 ions/nm^2^ and with an energy of 32 keV), silicon in the ion path becomes fully amorphous, forming nanobubbles in the affected region. Due to the low solubility of helium in amorphous silicon, the implanted helium becomes trapped beneath the surface, causing the amorphous layer to deform into a balloon-like structure; the internal pressure continues to rise, stretching the silicon membrane until it eventually bursts [28].

The SEM image (Figure 1b) of the nano-spherical tip shows that it exhibits an ideal spherical surface with extremely low roughness ~ 0.2 nm [29], and the diameter of the nanosphere is controllable between 100 nm to 1 µm. The nano-spherical tip enhances spatial resolution compared with conventional micro-scale spherical tips, which is particularly beneficial when investigating nanomaterials with a modulus distribution on a micro-nano scale and biomaterials like cells, bacteria, DNA, or proteins. This improved resolution allows more precise measurement and meaningful mapping of their mechanical properties and interactions. Furthermore, compared with the spherical probes already available on the market, which are prepared by sub-micron particle attachment methods, the sphere and the cantilever of our novel tip are integral. They therefore offer more reliable and repeatable measurements [27,30].

To demonstrate the high specified resolution of the nano-spherical tip, we employed it to measure the adhesion force between silicon and glass. Figure 1c,d show an adhesive force map including 1024 points on a glass film and a statistical analysis of the data. It was found that the adhesive force obeyed the Gaussian distribution well; the detailed statistical information is in Table S1. This experiment proves that the novel nano-spherical tip is stable when measuring surface adhesion force with high spatial resolution [27].

**Figure 2:**
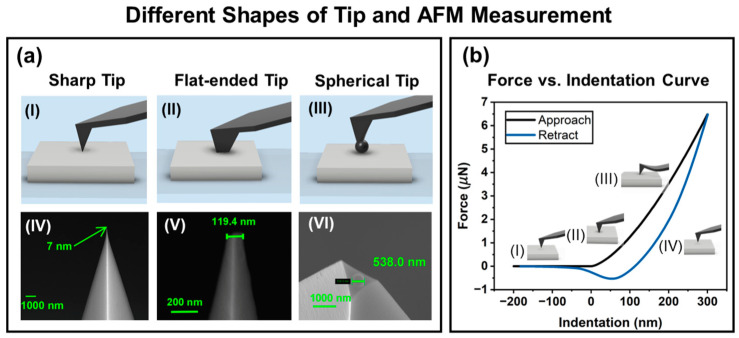
Different shapes of tip and AFM measurement: (**a**) three different tips: (**I**) schematic of the sharp tip; (**II**) schematic of the flat-ended tip; (**III**) schematic of the spherical tip; (**IV**) SEM image of the sharp tip; (**V**) SEM image of the flat-ended tip; (**VI**) SEM image of the spherical tip; (**b**) schematic of the AFM measurement process: (**I**) approach; (**II**) contact; (**III**) deformation; (**IV**) retraction.

To investigate the measurement accuracy and precision of different types of tips, force–indentation curves were obtained from tests on two polymers and fitted with the Oliver–Pharr and Hertz models, respectively. The schematics of the experiment and SEM images of the tips used are shown in Figure 2. Furthermore, this research also explored tip wear through an experiment examining the relationship between the radius of the blunted tip and the scanning length, applying flat-ended tips as a set of references for comparison. Our findings advocate for the nano-spherical tip, which exhibited minimal variation and represents the more precise option for measuring the elastic modulus of polymer materials.

## 2. Materials and Methods

### 2.1. Materials

A single-crystal silicon (100) piece approximately 1 cm × 1 cm size was utilized for performing the tip wear experiment. It was firstly cleaned with acetone, IPA, ethanol, and distilled water and then blown dry using nitrogen gas. The silicon piece was firmly glued onto a glass slide for the tip scanning wear test.

Polytetrafluoroethylene (PTFE) and polypropylene (PP) were used for the elastic modulus measurement. PTFE is widely recognized under the trade name Teflon and is a fluoropolymer renowned for its distinctive characteristics. These properties encompass non-reactivity, hydrophobicity, a low coefficient of friction, and excellent insulating capabilities. Predominantly employed as a nonstick coating in cookware, PTFE has found extensive applications in various industries, including semiconductor manufacturing and the production of medical devices [31]. PP is the main output of propylene, among other derivatives, with a two-thirds consumption rate. It has a density of 0.90 g/cm^3^ and is the lightest type of plastic. Homo-polymer PP (HPP) has a 65–75% market share. Branching, reinforcing, and filling PP are some techniques to produce plastics with superior mechanical properties [32]. The Poisson’s ratio used in the Hertz model is 0.46, according to Professional Plastic, attached as Table S2.

First, the two materials intended for testing were carefully cut into small pieces, each measuring roughly 10 mm × 10 mm. Following this, the surfaces of the pieces were meticulously cleaned using ethanol and left to air dry. Once dry, the materials were securely glued onto the substrate using double-sided tape, ensuring firm adhesion throughout the testing process. The surface topography of the two materials is shown in Figure S1 in the Supplementary Materials. The roughness of the material surface can significantly affect elastic modulus measurements [33]. The root mean roughness of the surface measured by AFM was 55.21 nm for PTFE and 152.464 nm for PP, within the reasonable range referring to the values reported in the literature (PTFE, 0.25 nm–5.23 μm [34,35]; PP, 6 nm–380 nm [36,37,38]). The two materials underwent nanoindentation testing (Anton Paar, UNHT3) in this experiment to acquire more conventional values. These obtained values serve as initial estimates in the subsequent process of fitting the force–indentation curve using the Hertz model. The outcomes are presented below in Table 1 for comparison and analysis.

Three different shapes of tips were used to test the elastic modulus of materials. All of them were made by the Olympus Company (Oxford, UK). The specific model was AC160 TS-R3. The material was silicon with aluminum coating, and the radius was 7 nm. The frequency of the lever was 300 (200–400) kHz, and the spring constant k was equal to 26 (8.4–57) N/m. The real spring constant of each cantilever was calibrated and stored for further calculation. The average value of all the tips used was 27.05 N/m. Detailed information on the spring constant of each tip is listed in Table S3.

The tetrahedral tip was the original one without any modifications. The tip shape was three-sided with a front angle of 0°, a back angle of 35°, and a side angle of 15°. For ease of reading, tetrahedral tip is abbreviated to sharp tip in the rest of this paper. The flat-ended tip was fabricated by cutting the sharp tip with a focused ion beam, also known as FIB. SEM confirmed the dimensions of the flat-ended tip. The side length of the flat-ended tip was around 500 nm. To confirm the result, another smaller flat-ended tip with an approximate diameter of 100 nm was produced by blunting the sharp tip to a circular surface using contact scanning. SEM confirmed the exact tip size, as shown in Figure 2 (V). The nano-spherical tip was fabricated using the method invented by Hu et al. [27]. The technique used was Helium Ion Microcopy (HIM) based on Gas Field Ion Sources (GFISs). An ORION nanofab manufactured by Carl Zeiss (Oberkochen, Germany) was employed with an amplitude of 9.74 pA, a voltage of 30 kV, and a corresponding energy of 30 keV. The dose was 40,000 ions/nm^2^, the set diameter was 400 nm, and the dwelling time was 0.1 μs. The whole fabrication process was carried out within 5 min. The radii of the two nano-spherical tips used were 250.35 nm and 269.00 nm, confirmed by SEM.

### 2.2. Methodology

#### 2.2.1. AFM Measurements

All AFM measurements were performed using an Oxford Instruments (Oxford, UK) model MFP-3D-Origin+. Force–indentation curves were obtained using force mapping in the contact mode. The cantilever was calibrated using the GetReal function [39] in AR Software (Version 28). The resonance frequency, deflection InvOLS, amplitude InvOLS, and spring constant were obtained via this process.

The force mapping mode was applied to the two materials to obtain 60 sets of data for each tip. Every test was conducted in the same position but with unavoidable slight displacement due to changing different tips. The maximum force used was controlled at approximately 700 nN for both materials. The total indentation depth was controlled at 200 nm. The data used for the Hertz model fitting were within 50 nm and the maximum force was around 200 nN. After obtaining the deflection vs. indentation depth curve, the software automatically converted the displacement data into force value using parameters that had been calibrated and stored previously.

An AFM experiment consists of two distinct stages, each exhibiting a unique force curve. The initial stage is known as the “approach curve”, during which the tip and the sample approach each other until contact is made. Subsequently, the “retract curve” follows, as the tip withdraws from the sample, potentially revealing adhesion forces. In Figure 2, the black line represents the extension process, and the blue line represents the retraction process. The tip first approaches the substrate (I) and then makes contact with the sample (II), and the cantilever performs a deformation during the indentation progress (III). Then, the cantilever begins to retract (IV). Due to the adhesion force and elastic–plastic deformations, the retraction curve is not the same as the extension curve.

#### 2.2.2. Data Processing

Here, the adhesion force is relatively small (Load_max_/F_adhesion_ > 10) and can be ignored. For the spherical tip, since the contact area is relatively large, the pressure is evenly distributed, so the elastic–plastic deformation can also be ignored. Thus, it is appropriate to fit the data using the Hertz model. For the sharp tip, as the radius is assumed to be 7 nm, the contact area is relatively small, and elastic–plastic deformation is prominent, so the data is fitted using the Oliver–Pharr model. 

The Hertz model was first put forward by Heinrich Hertz in 1882 [40] and is one of the most typical models applied to analyze two-surface contact for AFM and elastic modulus measurement [41]. This model ignores the relatively low adhesion forces and can be applied to a spherical contact surface and a flat substrate [42]. The Hertz theory’s final equations, which relate the contact area’s size, contact pressure, and elastic compression to the load, geometry, and elastic moduli, often appear to the practicing engineer as a magic formula.

During indentation, a compression force *P* is applied to the surface and the displacement *h* is measured. The relation between the two variables is as follows [43,44]:

(1) $$\frac{dP}{dh}=2\cdot E^{*}\cdot r_{c}$$

where $E^{*}$ is the reduced elastic modulus, and $r_{c}$ is the contact radius. If the tip is flat-ended, then the radius of contact does not change during the process of indentation. The applied force *P* and indentation depth *h* have the following relationship:

(2) $$P=E^{*}\cdot 2R\cdot h\;$$

where $E^{*}$ is the reduced elastic modulus, *R* is the curvature radius of the tip, and *h* is the indentation depth.

If the tip is spherical, the Hertz model leads to the following dependence of *P* on *h*:

(3) $$P=\frac{4}{3}\cdot E^{*}\cdot R^{\frac{1}{2}}\cdot h^{\frac{3}{2}}$$

Considering the deformation of the silicon tip, the elastic modulus of sample can be calculated as follows:

(4) $$\frac{1}{E^{*}}=\frac{1-v_{t}^{2}}{E_{t}}+\frac{1-v_{s}^{2}}{E_{s}}\;$$

where $E_{t}$ and $E_{s}$ are the elastic moduli and $v_{t}$ and $v_{s}$ are the Poisson’s ratios of the tip and the specimen, respectively. $E^{*}$ can be determined from the curve fitting using Equations (2) and (3).

**Figure 3:**
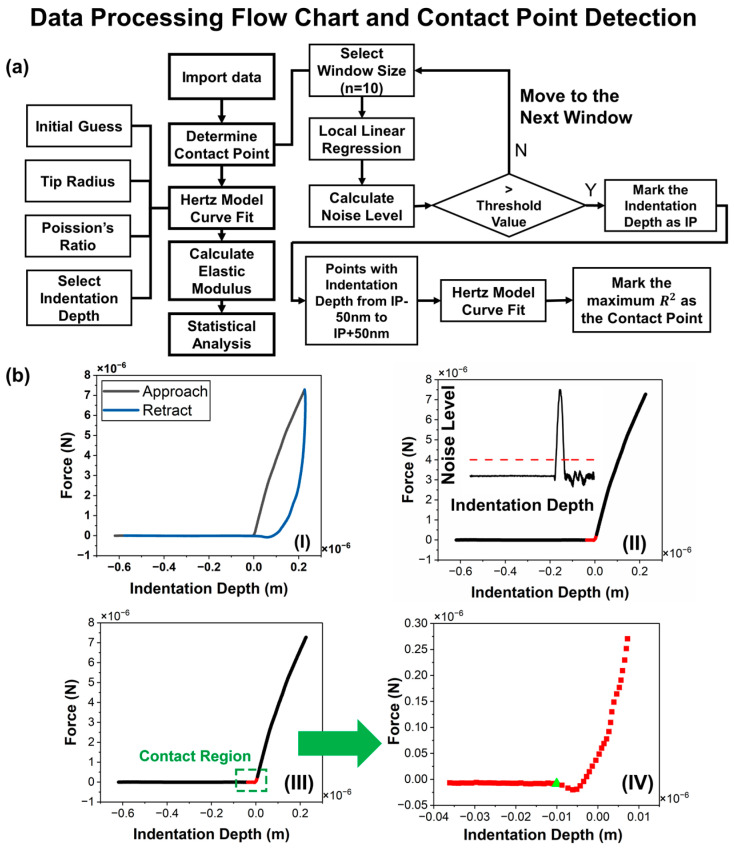
(**a**) Data processing flow chart. (**b**) Contact point detection: (**I**) the raw force vs. indentation depth curve. The black curve represents the approach process, and the blue curve represents the retracting process; (**II**) the noise level of the data computed locally; (**III**) the contact region within the green dashed box, identified starting from where the noise level is higher than the threshold; (**IV**) The red curve represents the magnified contact region, and the green point represents the contact point determined by a set of fit curve.

When analyzing the force vs. indentation depth data, it is very important to detect the contact point of the curve, since this point affects the fitting curve and hence the accuracy of calculating the elastic modulus of the material [45]. As shown in Figure 3, a self-written program determines the contact point via a combination of the two methods and then applies curve fitting to minimize the difference between the observed data points and the values predicted by the model. Two methods [45,46] of detecting the contact point are integrated and innovated to locate the contact point. First, the contact region needs to be determined appropriately. So, the algorithm is designed to trigger two alarms, tracking the deviation of the approach curve. The region between the two alarms is designated as the contact region [46]. Then, a set of fitting curves is used to fit every point in the contact region, and the R-squared values are calculated. The point where the fitting curve gives the highest R-squared value is the point that is recognized as the contact point [45]. In this experimental data processing, the elastic modulus obtained from nanoindentation was used as the initial guess and the local optimal elastic modulus that fitted the Hertz model was obtained. 

Introduced in 1992, the Oliver–Pharr method for measuring hardness and elastic modulus through instrumented indentation techniques has gained widespread adoption and is extensively used to characterize small-scale mechanical behavior [47,48]. This approach considers the elastic–plastic deformation that occurs at the contact interface between the indenter tip and the material, enabling a more precise evaluation of mechanical properties [16].

Since it is inappropriate to neglect the elastic–plastic deformation caused by the sharp tip, we have adopted the Oliver–Pharr model here, applying the following procedure for the calculations.

The unloading curves have approximately the following relation [47,48]:

(5) $$P=\alpha {\left(h-h_{f}\right)}^{m}\;$$

(6) $$S={\left.\frac{dP}{dh}\right|}_{h=h_{max}}$$

where P is the unloading load, h is the unloading depth, $h_{f}$ is the final depth, α and m are fitting parameters, and *S* is the unloading stiffness. Although Oliver [48] reported values of the fitting parameters observed with a Berkovich indenter, these are not suitable for fitting the unloading curve obtained by AFM. Thus, based on Equation (5), a self-written Python (Spyder, Version 5) program for linear regression analysis was run to fit 50 datasets near the maximum load with displacement of the unloading data to avoid the complexity related to Equation (6).

(7) $$S=\frac{n\sum hP-\sum h\sum P}{n\sum h^{2}-{\left(\sum h\right)}^{2}}$$

where *n* is 50, and *h* and *P* are data near the maximum displacement and unloading load. The $R^{2}$ for linear regression is also calculated as below:

(8) $$R^{2}=1-\frac{\sum {\left(P-\hat{P}\right)}^{2}}{\sum {\left(P-\bar{P}\right)}^{2}}$$

where $\hat{P}$ represents the predicted unloading values and $\bar{P}$ is the mean of the actual unloading values. The value of $R^{2}$ is equal to 0.98 for both materials.

(9) $$E^{*}=\frac{\sqrt{\pi }}{2}\frac{S}{\sqrt{A}}$$

where A is the contact area, calculated as below:

(10) $$A=\pi \tan^{2}\theta h_{c}^{2}\;$$

where θ is the half angle of the conical tip, assumed to be $35^{{}^{\circ}}$ based on the back angle of the three-sided pyramidal tip, and $h_{c}$ is contact depth.

(11) $$h_{c}=h_{max}-\epsilon \frac{P_{max}}{S}\;$$

where $P_{max}$ is the maximum load, where ϵ is calculated as 0.72 for the conical indenter.

(12) $$\epsilon =\frac{\pi }{2}\left(\pi -2\right)$$

Elastic modulus *E* was calculated using Equation (4). Statistical analyses were performed on all the elastic modulus calculations above. Detailed results are presented in the next section.

## 3. Results and Discussion

**Figure 4:**
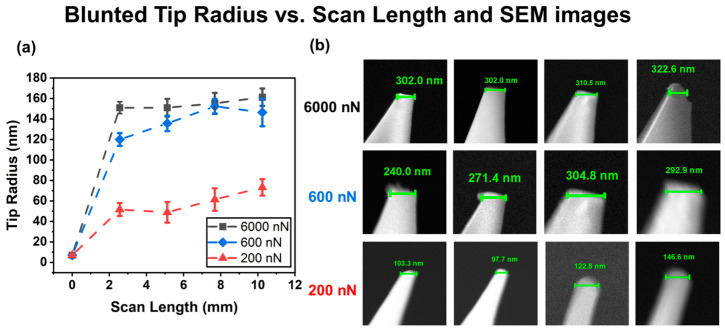
(**a**) Blunted tip radius vs. scan length and SEM images. (**b**) In the SEM images of the tips, the first line indicates an applied force of 6000 nN, the second line an applied force of 600 nN, and the third line an applied force of 200 nN. From left to right, the scan distance increased uniformly from 0 mm to 10.24 mm, with 2.56 mm spacing.

The research aimed to investigate the precision of nano-spherical tips and to analyze the sources of errors for the sharp tips and flat-ended tips when measuring the elastic modulus. A sharp tip can be worn out during contact scanning. To investigate the wearing-out process, contact scanning was applied with different scales of forces (200 nN, 600 nN, and 6000 nN), such as are commonly used in AFM mechanical properties measurements of polymers [14,49,50,51]. The scanning area was 5 µm × 5 µm, with 256 lines per image. Each image required the tip to scan 2.56 mm in length. The tip radius was measured using the built-in scale of the software under the SEM images, with the specific starting position manually selected. Figure 4a shows the median tip radius of three measurements versus scanning length. The corresponding SEM figures are shown in Figure 4b.

First, it was observed that the tip radius initially increased quickly and then increased slower and eventually saturated. The initial fast increase was attributed to the high contact stress on the sharp tip resulting in a large wear-out rate. As the tip size increased, the contact stress was reduced and the wear-out rate decreased. Therefore, the tip wear-out rate fell. Second, when applying a smaller normal force, the tip radius increased and was saturated to a smaller tip size; 200 nN resulted in a tip radius of 73.3 nm while 6000 nN resulted in a tip radius of 161.8 nm, which is reasonable because a smaller applied force generates smaller contact stress resulting in a smaller wear rate.

The sharp tip became worn out during the contact scanning and the actual tip radius could not be measured directly since the tip radius was beyond the detection limit of a normal optical microscope; thus, it was impossible to check accurately the size of the tip without using SEM, which would have been impractical to implement. There are various complicated methods to determine the tip radius as well as the contact area. The geometrical model method was developed using AFM to scan a step structure, assuming the tip had a hemispherical cone shape. Fast Fourier transform (FFT) was used to simulate profiles from tips with varying radii [52]. The AFM tip characterizer utilizes comb-shaped lines and gratings to measure probe dimensions with high precision and evaluate a range of probe sizes [53]. The calibration grating scanning method involves scanning a standard grating to derive the probe’s curvature radius using a nonlinear regression function [54]. The single force–indentation curve method estimates the tip radius by fitting the curve’s tangent at maximum indentation depth using related equations [55]. However, these methods do not provide a direct way to obtain the tip characteristics. They often require complex procedures such as curve fitting, nonlinear regression, and signal processing techniques like FFT, which involve multiple parameters and time-consuming analysis. Additionally, as sharp tips wear down over time, these determination methods need to be applied frequently to ensure the accuracy of the tip shape.

Different contact models consider the effect of tip geometry on the calculation of the elastic modulus [56,57,58]; however, they all require precise estimation of the contact area. For example, in the Oliver–Pharr model and Sneddon model, the half angle of the conical tip is the crucial factor for deriving the elastic modulus based on force–indentation curves. Due to tip wear, contamination, and surface roughness, the half angle varies and the contact area changes as the force contact mode is applied. Since there is no direct method to obtain the tip geometry accurately, large variations in AFM modulus measurement have commonly been reported [43,45,59]. In addition to mechanical wear due to tip scanning, tip fracture may also be observed, leading to a more dramatic change in tip size, as shown in Figure S2 in the Supplementary Materials.

**Figure 5:**
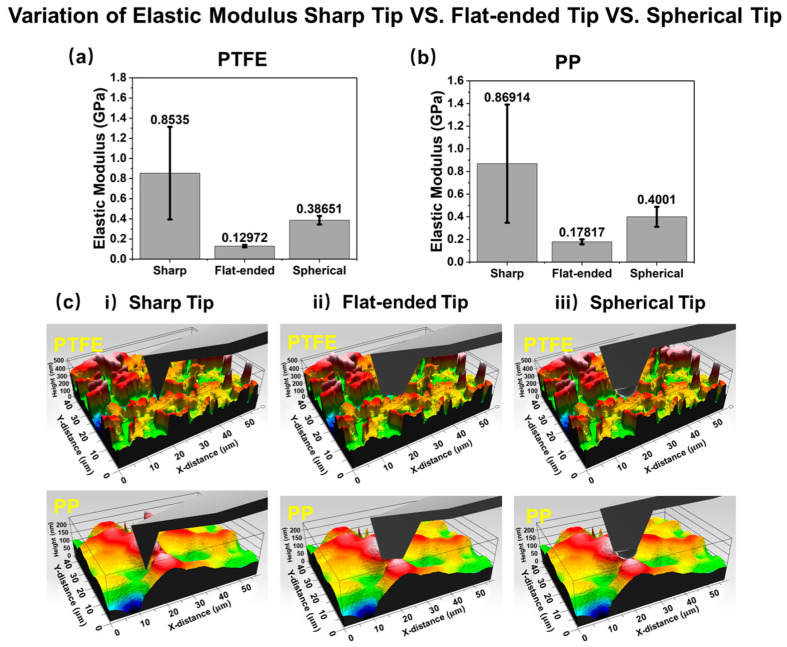
Variation of elastic modulus in the sharp tip vs. flat-ended tip vs. spherical tip: (**a**) PTFE; (**b**) PP; (**c**) schematics of the three tips in contact with the surfaces of the two materials, which were characterized via a 3D laser scan. Colors in the images represent variations in height.

To consider the case of a flat-ended tip with a relatively constant contact area, two flat-ended tips with a radius of around 50 nm and 250 nm were applied to test the same material. Assuming the spherical contact not to be rigorous from the geometric perspective since the contact surface was flat, the data were fitted for the flat-ended contact mechanism (Equation (2)). The elastic modulus values measured by the flat-ended tip with a radius of 50 nm are shown in Table 2. The results of the measurement of the elastic modulus of both flat-ended tips were relatively very small, far from the reasonable range. These relatively very small values may have resulted from a slight angle between the tip and sample surface due to the nano-scale roughness of the sample surface, demonstrated in Figure 5c. So, the contact area was lower, and the indentation depth was deeper than they were assumed to be. Therefore, since the exact contact mechanism is unknown, the flat-ended tip results should be considered unreliable when the goal is to obtain an accurate elastic modulus.

Since the experiment already demonstrated that the sharp tip would be blunted and the flat-ended tip would encounter the problems of vague contact conditions, to improve the precision and accuracy of the elastic modulus measured by AFM, a new technology can be applied to manufacture nano-spherical probes that have a defined radius and a symmetrical semi-spherical geometric shape that fits the Hertz model and hardly ever wears out during the test process, resulting in less error and more reliable data results. Experiments and data have confirmed that this novel nano-spherical tip can improve the precision of elastic modulus measurements. The specific data and comparison are shown in the following paragraphs.

Upon comparing the three datasets, it was evident that the variation observed with the spherical tip is significantly smaller compared with that for the sharp tips fitted with the appropriate model. Consequently, it is recommended to employ the spherical tip for measuring the elastic modulus of soft polymer materials. Moreover, the elastic modulus measured by the spherical tip and sharp tip was lower than that measured by the nanoindentation method, which has also been seen in other research [15,49,60]. This may be because AFM is more sensitive to surface characteristics while nanoindentation measurements average out the effects of surface roughness and other local defects, providing a more representative measure of the material’s overall elastic modulus.

As analyzed above, commercial sharp tips are prone to blunting during indentation, making it difficult to accurately estimate the real contact area of the tip, which is a crucial factor for processing the force–indentation data. Therefore, the fluctuation in tip contact conditions is identified as the primary cause for the substantial deviation in elastic modulus measurements. It has been observed that the elastic modulus values obtained from a flat-ended tip exhibit a relatively small magnitude. This phenomenon is attributed to misalignment between the probe and the sample, resulting in contact mechanism problems. If the contact mechanism is a flat surface, the force–indentation depth should exhibit a linear relationship, which was not seen in the case here [19], where the magnitude was much lower than the nano-indentation values. Therefore, it is concluded that flat-ended tips are not suitable for conducting elastic modulus tests via AFM. Research [33,61] suggests that higher roughness leads to less precision in the measurement of the elastic modulus. The imaging (Figure 5c) and surface roughness data obtained using 3D laser scanning (Filmetrics, Profilm3D, San Diego, CA, USA) serve to visually support the challenges faced by both sharp and flat tips in terms of uncertain contact conditions. A scheme (Figure S3) is further used to illustrate the contact conditions clearly.

## 4. Conclusions

When using AFM to measure the elastic modulus of a material, the precision of the results is highly dependent on the estimation of the contact area. However, due to the nanoscale nature of the material, the actual contact condition can be affected by many factors (tip wear, fracture, and sample morphology). Thus, it is difficult to accurately obtain the contact area. This experiment indicates that a sharp tip becomes blunted during contact measurement. The uncertainty of the tip radius leads to huge variations when using the Hertz model to fit the force–indentation data. Another option is the flat-ended tip, which has a relatively constant contact area, but the results showed that when a flat-ended mechanism was applied to fit the force–indentation data, the elastic modulus was significantly lower than the reasonable range. Thus, flat-ended tips cannot provide accurate values of mechanical properties, but they can still be applied to investigate the relationship between the modulus of elasticity and changes in external factors. The newly invented nano-spherical tip is optimal for measuring soft materials’ elastic modulus, especially when it fits the force–indentation data with the Hertz model. This is because the spherical tip maintains a clear and relatively constant tip radius during the contact process and can avoid misalignment between the tip and the substrate surface, due to its symmetry structure. In essence, while AFM presents outstanding capabilities for nano-scale mechanical property analysis, the precision of the results is affected by the tip geometry. Applying this novel nano-spherical tip will yield more precise and reliable results.

Looking ahead, the nano-spherical tip presents significant potential for applications such as manipulation and interface cleaning. Firstly, it demonstrates high efficiency in removing bubbles and contaminants from 2D material heterostructures, thereby improving the quality of these interfaces [62]. Secondly, compared with conventional sharp tips, the nano-spherical tip induces less mechanical damage during the manipulation of nanoparticles via atomic force microscopy, as sharp tips can cause mechanical degradation when used for contact scanning and manipulating nanoparticles [63,64].

## Figures and Tables

**Table 1:** Elastic modulus of PTFE and PE (nanoindentation).

Material	Elastic Modulus (GPa)	Standard Deviation
PTFE	1.862	0.092
PP	0.850	0.224

**Table 2:** Variation of Elastic Modulus in Three Types of Tips.

Elastic Modulus (GPa)	PTFE			PP		
Tip	Mean	SD	RSD	Mean	SD	RSD
Sharp Flat-ended Spherical	0.85919 0.12972 0.38651	0.4857 0.0122 0.0423	0.565 0.144 0.109	0.85350 0.17817 0.40010	0.45970 0.0217 0.0884	0.539 0.124 0.211

## Data Availability

The original contributions presented in the study are included in the article, further inquiries can be directed to the corresponding authors.

## Supplementary Materials

The following supporting information can be downloaded at: https://www.mdpi.com/article/10.3390/mi15091175/s1, Figure S1: the topography of PP and PTFE; Figure S2: tip fracture; Figure S3: scheme of contact situations; Table S1: statistical information of the nano-spherical probe in adhesion force measurement; Table S2: Poisson’s ratio reference data from Professional Plastic; Table S3: spring constant of each tip.
