# Supplementary material for: A Novel Nano-Spherical Tip for Improving Precision in Elastic Modulus Measurements of Polymer Materials via Atomic Force Microscopy
Source: Micromachines (Basel). 2024 Sep 22;15(9):1175. doi: 10.3390/mi15091175 (PMC11434511; doi:10.3390/mi15091175)
Supplement: Supplementary file 1 [file micromachines-15-01175-s001.zip › micromachines-3172383-supplementary.pdf]

Supplementary Materials

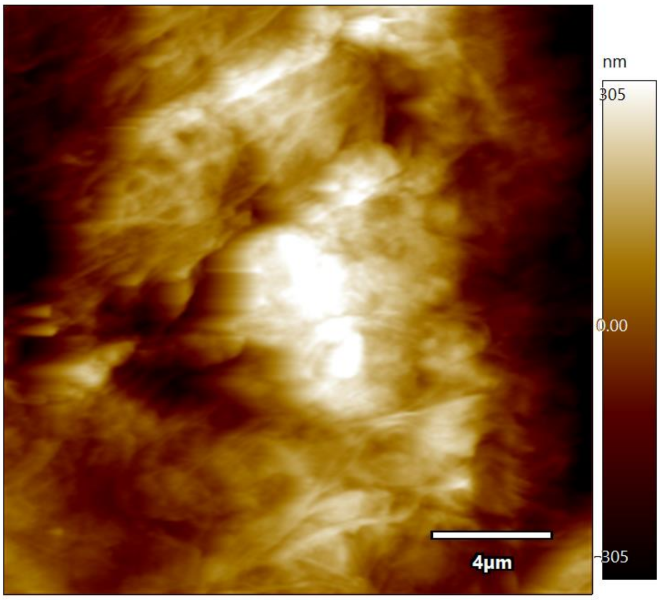

Figure S1 a): the topography of PP

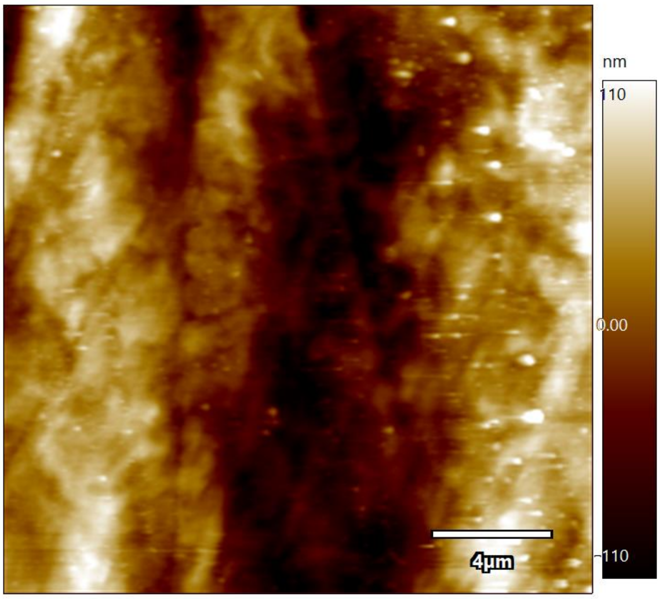

Figure S1 b): the topography of PTFE

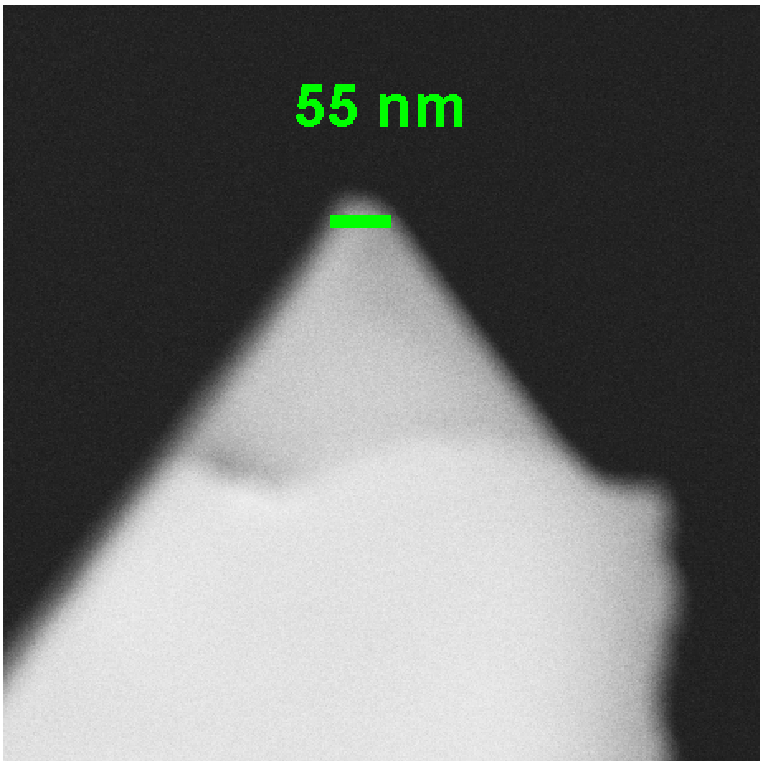

Figure S2: tip fracture

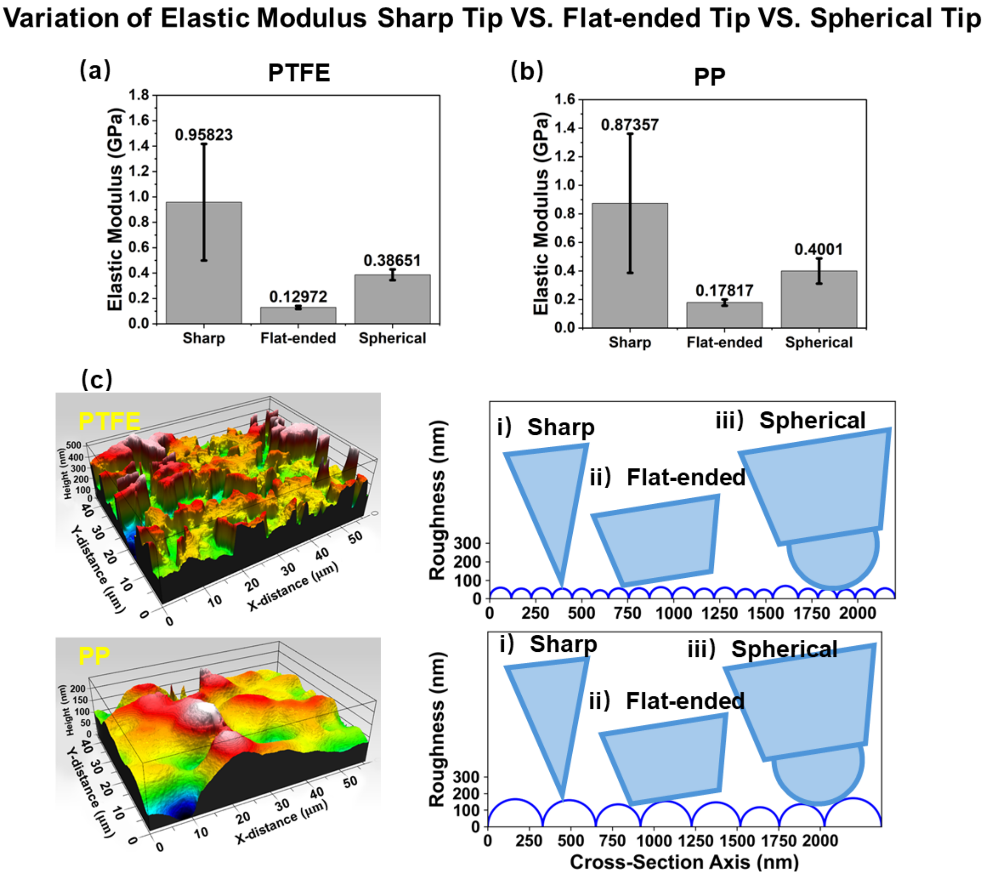

Figure S3: scheme of contact situations

Table S1: statistical information of the nano-spherical probe in adhesion force measurement

Nonlinear Curve Fit (Gauss) (2024/7/23 21:42:12)

Parameters

		Value	Standard Error	t-Value	Prob>|t|	Dependency
Count	y0	3.43115	2.8075	1.22214	0.24183	0.61927
	xc	2.6127E-7	6.53922E-10	399.54305	8.35379E-30	1.7793E-4
	w	4.36496E-8	1.60934E-9	27.12264	1.67356E-13	0.55831
	A	9.65442E-6	4.06289E-7	23.76245	1.0274E-12	0.74681
	sigma	2.18248E-8	8.0467E-10			
	FWHM	5.13934E-8	1.89485E-9			
	Height	176.4763	5.029			

Reduced Chi-sqr = 54.0174340593
COD(R^2) = 0.9891633495977
Iterations Performed = 5
Total Iterations in Session = 5
Fit converged. Chi-Sqr tolerance value of 1E-9 was reached.
Standard Error was scaled with square root of reduced Chi-Sqr.
sigma, FWHM, Height are derived parameter(s).

Statistics

	Count
Number of Points	18
Degrees of Freedom	14
Reduced Chi-Sqr	54.01743
Residual Sum of Squares	756.24408
R-Square (COD)	0.98916
Adj. R-Square	0.98684
Fit Status	Succeeded(100)

Fit Status Code :
100 : Fit converged. Chi-Sqr tolerance value of 1E-9 was reached.

Summary

	y0		xc		w		A		sigma		FWHM		Height		Statistics	
	Value	Standard Error	Value	Standard Error	Value	Standard Error	Value	Standard Error	Value	Standard Error	Value	Standard Error	Value	Standard Error	Reduced Chi-Sqr	Adj. R-Square
Count	3.43115	2.8075	2.6127E-7	6.53922E-10	4.36496E-8	1.60934E-9	9.65442E-6	4.06289E-7	2.18248E-8	8.0467E-10	5.13934E-8	1.89485E-9	176.4763	5.029	54.01743	0.98684

ANOVA

		DF	Sum of Squares	Mean Square	F Value	Prob>F
Count	Regression	3	69029.5337	23009.84457	425.97071	<0.0001
	Residual	14	756.24408	54.01743		
	Uncorrected Total	18	128040			
	Corrected Total	17	69785.77778			

Count: At the 0.05 level, the fitting function is significantly better than the function y=constant.

Fitted Curves Plot

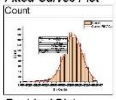

Residual Plots

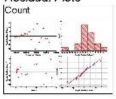

Table S2: Poisson's ratio reference data from Professional Plastic

Professional Plastics

The Global Leader in High Performance Plastics

Mechanical Properties of Plastic Materials

Material	Formula	Abrasive resistance - ASTM D1044 mg/1000 cycles	Coefficient of friction	Compression set after 24 hr at 175C %	Compressive modulus GPa	Compressive strength MPa	Elongation at break %	Hardness - Rockwell	Izod impact strength J m'	Poisson's ratio	Tear strength N mm'	Tensile modulus GPa	Tensile strength MPa
**Cellulose Acetate Butyrate**	CAB	-	-	-	-	-	60	99	260	-	-	0.3-2.0	20-60
**Ethylene-Chlorotrifluoroethylene copolymer**	E-CTFE	5	0.07-0.08	-	-	-	200	R95	<1000	-	-	1.4-1.6	48
**Ethylene-Tetrafluoroethylene Copolymer**	ETFE	-	-	-	-	-	250-350	R50	>1000	-	-	0.8	28-48
**Fluorinated Ethylene Propylene Copolymer**	FEP	-	0.27-0.67	-	-	-	150-300	R25-45, 60 Shore D	No break	0.48	-	0.5 - 0.6	14 - 30
**Polyacrylonitrile-butadiene-styrene**	ABS	-	0.5	-	-	-	45	R100-110	200-400	0.35	-	2.1-2.4	41-45
**Polyamide - Nylon 6**	PA 6	5	0.2-0.3	-	-	-	-	M82	30-250	0.39	-	2.6-3.0	78
**Polyamide - Nylon 6, 6**	PA 6,6	3-5	0.2-0.3	-	-	-	-	M89	40-110	0.41	-	3.3	82
**Polyamide - Nylon 6, 6 - 30% Carbon Fiber Reinforced**	PA 6, 6 - 30% CFR	-	-	-	-	-	2	-	-	-	-	24	260

Material	Formula	Abrasive resistance - ASTM D1044 mg/1000 cycles	Coefficient of friction	Compression set after 24 hr at 175C %	Compressive modulus GPa	Compressive strength MPa	Elongation at break %	Hardness - Rockwell	Izod impact strength $J\ m^{-1}$	Poisson's ratio	Tear strength $N\ mm^{-1}$	Tensile modulus GPa	Tensile strength MPa
**Polypropylene**	PP	13-16	0.1-0.3	-	-	-	150-300, for biax film >50	R80-100	20-100	-	-	0.9-1.5, for biax film 2.2-4.2,	25-40, for biax film 130-300,
**Polystyrene**	PS	-	-	-	-	-	1.6	M60-90	19-24	0.35	-	2.3-4.1	30-100
**Polystyrene - Conductive**	High Impact Conductive Polystyrene	-	-	-	-	-	36	-	no break	-	-	1.6	27
**Polystyrene - Cross-linked**	PS - X - Linked	60-100	-	-	-	-	3-5	R110-120	-	-	-	1.65	55-70
**Polysulphone**	PSu	-	-	-	-	-	50-100	M91	69	-	-	2.48	70
**Polytetrafluoroethylene**	PTFE	-	0.05-0.2	-	-	-	400	D50-55 - Shore	160	0.46	-	0.3-0.8	10-40
**Polytetrafluoroethylene filled with Glass**	PTFE 25% GF	-	0.08-0.10	-	-	-	100-300	D60-70 - Shore	144	-	-	1.7	7-20
**Polyvinylchloride - Unplasticized**	UPVC	-	-	-	-	-	60	R106-120	20-1000	-	-	2.5-4.0	25-70
**Polyvinylfluoride**	PVF	-	-	-	-	-	90-250	D80 - Shore	180	0.4	130-200 (initial)	2.1 - 2.6	55-110
**Polyvinylidenefluoride**	PVDF	24	0.2-0.4	-	-	-	50	R77-83	120-320	0.34	-	1.0-3.0	25-60
**Silicone Elastomer**	MQ /VNQ /PMQ /P VMQ	-	-	-	-	-	-	60 degrees Shore A	-	-	-	-	6.5
**Tetrafluoroethylene-perfluoro(alkoxy vinyl ether) - Copolymer**	PFA. Teflon PFA.	-	-	-	-	-	300	-	-	-	-	-	25

All information and technical data are given as a guide only. Although every effort has been made to ensure that the information is correct, no warranty is given as to its completeness or accuracy.

Call Professional Plastics at (888) 995-7767 or E-Mail sales@proplas.com Order Online at www.professionalplastics.com

Table S3: spring constant of each tip

Table S3. Spring Constant of AFM tips (N/m)		
	Tip Geometry	
Materials	Tetrahedral	Spherical
PTFE	25.96	25.89
PP	26.68	28.55
Sizes	Flat-ended	
250 nm	29.03	
50 nm	26.21	
